# Social Support and Loneliness in Healthy Elders and Cognitive Impairment

**DOI:** 10.1002/alz70858_107810

**Published:** 2025-12-25

**Authors:** Kavya K Kumar, Aparna N P, Jyothi M S Gowda, Apurva Mittal, Himanshu Joshi, Subhashini K Rangarajan, Preeti Sinha, Sivakumar PT, Mathew Varghese, Ganesan Venkatasubramanian, Paul M. Thompson, John P John

**Affiliations:** ^1^ National Institute of Mental Health and Neurosciences (NIMHANS), Bengaluru, Karnataka, India; ^2^ Multimodal Brain Image Analysis Laboratory (MBIAL), National Institute of Mental Health and Neurosciences (NIMHANS), Bengaluru, Karnataka, India; ^3^ Departmet of Psychiatry, National Institute of Mental Health and Neurosciences (NIMHANS), Bengaluru, Karnataka, India; ^4^ St John's Medical College, Bengaluru, Karnataka, India; ^5^ National Institute of Mental Health and Neurosciences, Bengaluru, India; ^6^ Department of Clinical Neurosciences, NIMHANS, Bengaluru, India; ^7^ University of Southern California, Los Angeles, CA, USA; ^8^ Imaging Genetics Center, Mark and Mary Stevens Neuroimaging & Informatics Institute, University of Southern California, Marina del Rey, CA, USA; ^9^ Imaging Genetics Center, Mark and Mary Stevens Neuroimaging and Informatics Institute, Keck School of Medicine, University of Southern California, Marina del Rey, CA, USA; ^10^ Keck School of Medicine, University of Southern California, Los Angeles, CA, USA; ^11^ Multimodal Brain Image Analysis Laboratory, Centre for Brain Mapping & ADBS Neuroimaging Centre, Department of Psychiatry, National Institute of Mental Health and Neurosciences (NIMHANS), Bengaluru, Karnataka, India; ^12^ Multimodal Brain Image Analysis Laboratory, National Institute of Mental Health and Neurosciences (NIMHANS), Bengaluru, India

## Abstract

**Background:**

The 2024 Lancet report on dementia prevention emphasizes the impact of social disadvantages (e.g., low education, social isolation, low socioeconomic status) on cognitive decline risk. Growing evidence connects social health to cognitive status in older adults. This study explored social support and loneliness in individuals with Alzheimer's dementia (AD), mild cognitive impairment (MCI), and healthy elderly (HE) participants from the NIH/NIA‐funded India ENIGMA Initiative for Global Ageing and Mental Health (R01AG060610).

**Method:**

Data from 85 participants were analysed, including 47 HE, 24 MCI, and 14 AD, based on the Clinical Dementia Rating (CDR). Social support was assessed using the Lubben Social Network Scale (LSNS‐6), with <12 indicating social isolation risk. Loneliness was measured using the UCLA 3‐Item Loneliness Scale. Descriptive statistics were used for continuous and categorical variables, with chi‐square and Fisher's exact test to assess group associations.

**Result:**

The groups showed no significant age differences (*p* = 0.07). Most participants were male and lived in urban areas. HE had higher education (36.2% graduates), higher marriage rates (87.2%), and more retirees (59.6%) compared to MCI (37.5% graduates, all married, 75% retired) and AD (education evenly distributed, 57.1% married, 28.6% retired). Living arrangements varied significantly (*p* = 0.014), with most living with heir spouse and children. Significant differences were found in gender (χ^2^ = 8.6, df = 2, *p* = 0.13), marital status (*p* = 0.002), and employment (*p* = 0.014).

Social networking (LSNS) was stronger in HE (60.4%), while MCI and AD had equal proportions (50%) of strong and weak networks, with no significant group association (χ^2^ = 0.9, df = 2, *p* = 0.63). Loneliness was reported by 16.7% of HE and AD, increasing to 25% in MCI, but was not significantly linked to cognitive decline (χ^2^ = 0.8, df = 2, *p* = 0.68).

**Conclusion:**

HE had stronger social ties and lower loneliness, while the MCI group experienced greater loneliness, likely due to reduced social participation. Similar social networks in MCI and AD suggest cognitive impairment affects relationships. Addressing loneliness and fostering social engagement are vital for well‐being in cognitively impaired older adults.

